# Model based on the automated AI-driven CT quantification is effective for the diagnosis of refractory Mycoplasma pneumoniae pneumonia

**DOI:** 10.1038/s41598-024-67255-8

**Published:** 2024-07-13

**Authors:** Yali Qian, Yunxi Tao, Lihui Wu, Changsheng Zhou, Feng Liu, Shenglong Xu, Hongjun Miao, Xiucheng Gao, Xuhua Ge

**Affiliations:** 1 Department of Emergency/Critical Medicine, Children’s Hospital of Nanjing Medical University, Nanjing, Jiangsu China; 2 School of Pediatrics, Nanjing Medical University, Nanjing, Jiangsu China; 3 Department of Diagnostic Radiology, Jinling Hospital, Medical School of Nanjing University, Nanjing, Jiangsu China; 4 Department of Respiratory Medicine, Children’s Hospital of Nanjing Medical University, Nanjing, Jiangsu China; 5 Department of Radiology, Children’s Hospital of Nanjing Medical University, Nanjing, Jiangsu China

**Keywords:** Medical research, Risk factors

## Abstract

The prediction of refractory Mycoplasma pneumoniae pneumonia (RMPP) remains a clinically significant challenge. This study aimed to develop an early predictive model utilizing artificial intelligence (AI)-derived quantitative assessment of lung lesion extent on initial computed tomography (CT) scans and clinical indicators for RMPP in pediatric inpatients. A retrospective cohort study was conducted on patients with M. pneumoniae pneumonia (MP) admitted to the Children’s Hospital of Nanjing Medical University, China from January 2019 to December 2020. An early prediction model was developed by stratifying the patients with Mycoplasma pneumoniae pneumonia (MPP) into two cohorts according to the presence or absence of refractory pneumonia. A retrospective cohort of 126 children diagnosed with Mycoplasma pneumoniae pneumonia (MPP) was utilized as a training set, with 85 cases classified as RMPP. Subsequently, a prospective cohort comprising 54 MPP cases, including 37 instances of RMPP, was assembled as a validation set to assess the performance of the predictive model for RMPP from January to December 2021. We defined a constant Φ which can combine the volume and CT value of pulmonary lesions and be further used to calculate the logarithm of Φ to the base of 2 (Log_2_Φ). A clinical-imaging prediction model was then constructed utilizing Log_2_Φ and clinical characteristics. Performance was evaluated by the area under the receiver operating characteristic curve (AUC). The clinical model demonstrated AUC values of 0.810 and 0.782, while the imaging model showed AUC values of 0.764 and 0.769 in the training and test sets, respectively. The clinical-imaging model, incorporating Log_2_Φ, temperature(T), aspartate aminotransferase (AST), preadmission fever duration (PFD), and preadmission macrolides therapy duration (PMTD), achieved the highest AUC values of 0.897 and 0.895 in the training and test sets, respectively. A prognostic model developed through automated quantification of lung disease on CT scans, in conjunction with clinical data in MPP may be utilized for the early identification of RMPP.

## Introduction

MP is a prominent pathogen associated with community-acquired pneumonia (CAP) in children. MPP is responsible for 10–40% of lower respiratory tract infections in hospitalized children, with a pneumonia incidence rate of up to 50% in children over 5 years of age^1^. While MPP is usually considered a type of self-limiting disease, sometimes it can cause various pulmonary and extrapulmonary complications, including necrotizing pneumonia, bronchitis obliterans, encephalitis, arthritis, pericarditis, hemolytic anemia, and develop into serious life-threatening pneumonia^2,3^. Macrolides are considered the primary treatment option for Mycoplasma pneumoniae in pediatric patients. Despite treatment with macrolide antibiotics for 7 days or more, some patients may exhibit ongoing clinical and imaging progression, which is defined as RMPP^4^. The incidence of RMPP is on the rise due to the emergence of drug-resistant MP strains^5^. Consequently, early recognition and management of RMPP are crucial for optimizing patient outcomes. Traditionally, laboratory biomarkers serve as major models for the prediction of RMPP including CRP, LDH, ferritin, d-dimer, and IL-17A, and the AUC values derived from individual markers ranging from 0.7 to 0.8^6–8^. However, the prognosis of a disease cannot be accurately predicted by relying solely on a single indicator, and the results remain unsatisfactory.

A previous study showed the incidence of lung consolidation was found to be higher in the RMPP group compared to the CMPP group, suggesting that this factor may also contribute to the prediction of RMPP^9^. The amalgamation of clinical parameters and radiological features by models has been documented in the literature^10,11^. CT imaging plays a vital role in diagnosing and monitoring disease progression in patients with MPP by offering detailed visualization of lung tissue and adjacent structures involved in the disease process^12,13^. Weihong Lu et al. elevated CT scores manually in identifying RMPP and obtained an AUC of 0.781^14^. By combining lung CT characteristics with clinical indicators, a hybrid model could be developed to enhance the accuracy of RMPP prediction beyond that of existing models. Currently, the interpretation of CT scans for the MPP is typically performed manually by radiologists, leading to susceptibility to intra- and inter-observer variability, particularly during periods of high reporting volume. In these circumstances, the implementation of an automated technique for quantifying lung inflammation may prove beneficial in alleviating the burden on radiologists while potentially enhancing the accuracy and uniformity of diagnostic reporting.

Deep learning-based artificial intelligence (AI) has recently made tremendous progress and has made great achievements in the field of vision and image processing. Law Kumar Singh et al. propose an enhanced customized R2-ATT U-Net deep learning network for retinal blood vessel extraction, aiding in the early detection of eye diseases^15^. Similarly, Charu Bhardwaj et al. reported a deep-learning ensemble approach for the grading of diabetic retinopathy severity^16^. Several research-based AI algorithms have emerged as useful tools in terms of quantitative evaluation of pulmonary diseases. A previous study proposed a hybrid model (LSTMCNN) achieved 98.66% accuracy and an ensemble model using deep CNN achieved 99.78% accuracy for COVID-19 detection from chest X-ray images and CT images^11^. These AI-assisted models exhibit high accuracy and have justified their performance in clinical practice.

However, to our knowledge, associations between AI-derived CT features quantifying pneumonia lesions and the risk of RMPP in patients with MP infection have not yet been reported. If AI-derived features from CT at an early stage of MPP can be used to predict RMPP, they can be particularly beneficial because of their non-invasiveness and efficiency. The purpose of this study was to develop a model based on automated detection and quantification of chest CT images in conjunction with clinical parameters and evaluate its efficacy in the early diagnosis of RMPP.

## Methods

### Patients and groups

A retrospective cohort study was conducted on children hospitalized at the Children’s Hospital of Nanjing Medical University with MMP from January 2019 to December 2020. Validation of the findings was carried out using a prospective cohort from January to December 2021. The diagnosis of MP infection was established through the identification of positive laboratory test findings. (serum MP antibody ≥ 1:320, or serum MP antibody ≥ 1:160 and the MP polymerase chain reaction (PCR) positive, or MP antibody titer of the recovery phase and acute phase increased or decreased by 4 times or more). Nasopharyngeal aspirate/swab specimens were obtained within 24 h of admission. Exclusion criteria included: (1) patients with congenital disorders, metabolic disease, immunodeficiency disease, blood tumor disease, bronchopulmonary dysplasia, nervous system dysplasia, and epilepsy; (2) patients infected with other pathogens; (3) patients undergoing MPP recovery; (4) patients older than 18 years of age. Informed consents were obtained from all the guardians of the pediatric patients and the study was approved by the institutional ethics committee of the Children’s Hospital of Nanjing Medical University. The research followed the Helsinki Declaration^17^.

### Data collection and study variables

Diagnostic Criteria of MPP: (1) The history as well as findings on physical examination and a chest X-ray were in line with the diagnosis of CAP; (2) had positive results for MP. (3) hospitalized patients under 18 years old. RMPP is defined as a case with prolonged fever accompanied by deterioration of radiological findings despite appropriate management and treatment with a macrolide antibiotic for ≥ 7 days^18^. The demographics, clinical information, and laboratory data were collected from inpatient electronic medical records and analyzed. Peripheral blood samples were obtained within 24 h after admission for the determination of the C-reactive protein (CRP), white blood cells (WBC), hemoglobin (Hb), platelets (PLT), lactate dehydrogenase (LDH), neutrophil (NEUT), erythrocyte sedimentation rate (ESR), alanine aminotransferase (ALT), aspartate aminotransferase (AST), creatine phosphokinase isoenzyme (CK-MB), creatine kinase (CK), procalcitonin (PCT), Total Protein (TP) and albumin (ALB).

In addition, the following data were also recorded: the highest temperature on the first day of admission (T), heart rate (HR) and respiratory rate (R) on the day of admission, preadmission fever duration (PFD), preadmission cough duration (PCD), and preadmission macrolides therapy duration (PMTD).

### CT acquisition parameters

All patients diagnosed with MPP underwent imaging w with a 64-CT scanner (Toshiba or Philips Healthcare) within 48 h of diagnosis. The imaging protocol utilized exposure parameters of 110 kV scanning tube voltage, 100 mAs reference tube current, layer thickness 2.5 mm, maximum scanning layer thickness 10 mm, interval 2.5 mm, pitch 0.9, thin layer reconstruction layer thickness after scanning was 1.5 mm. The scanning range extended from the thoracic entrance to the base of the lungs.

### AI-based quantization of CT images and processing

**Figure 1:**
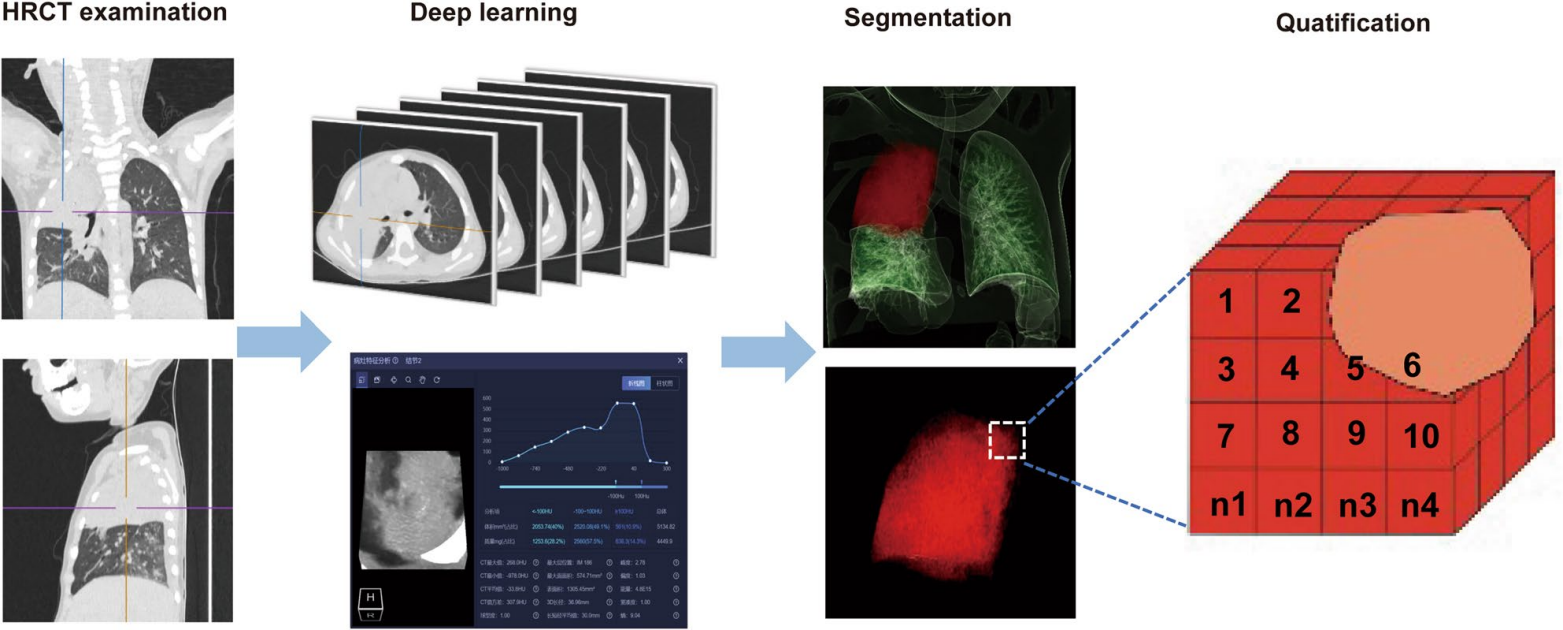
Pipeline for quantifying MP infection: The segmentation system based on deep learning is initially fed a chest CT scan. In the CT scan, quantifiable parameters are calculated to characterize infection areas, including but not limited to the following: infection volumes, and percentages of infection (POIs) in the whole lung, lung lobes, and bronchopulmonary segments. Finally, fine segment the 3D reconstructed lung image. 1 cubic centimeter (cm^3^) of the lesion was served as 1 minimum counting unit to calculate the CT value.

An AI-aided diagnosis system from Shenrui (Beijing, China) was used in this study. The segmentation of lung lobes, labeling of lung infections, and calculation of volumes were conducted using a commercially available deep-learning algorithm specifically designed for pulmonary pneumonia (Dr. Wise@Pneumonia, version 1.0, Beijing Deepwise & League of Ph.D. Technology Co., Ltd., China), which had been proved to be effective in the analysis of CT images from COVID-19 patients^19^. The system had three major modules. The first module was pneumonia lesion detection and segmentation, utilizing MVP-Net and 3D U-Net, with multi-view inputs and channel-wise attention mechanisms applied followed by multiple binary classifiers. The second module aimed to segment the pulmonary region into five lobes using an anatomical prior embedded network with a smooth margin loss. Finally, the Quantitative Evaluation module was able to calculate various metrics on top of the previously mentioned modules. Specific architecture details have been previously reported^19,20^. In general, the software is capable of automatically identifying the image characteristics of inflammatory lesions in chest CT images through the acquisition of a substantial dataset of CT images from pneumonia patients. It can then perform fully automated lesion delineation, measure lesion volume, and conduct quantitative analysis of volume proportions. Chest CT image analysis process: (1) Import the CT digital images from patients, and carry out 3D reconstruction of the whole lung, left and right lungs, lobes, and segments separately. (2) Segmentation of pulmonary lesions. (3) Count the total volume of the segmented lesions in the whole lung, left lung, and right lung separately. (4) The 3D reconstructed lung images underwent fine segmentation. 1 cubic centimeter (cm^3^) of lesion served as 1 minimum counting unit, the average density in this cubic centimeter was defined as ρ (ρ = HU + 1024, Hounsfield Unit, HU). (5) Finally, we define a constant Φ, Φ = ∑ (ρ1 + ρ2 + ρ3⋯ + ρ n-1 + ρn) (Fig. 1). (6) A logarithm of Φ to the base of 2 was used to analyze the data.

### Statistical analysis

Statistical analysis was performed using the R 4.04 version (R Foundation for Statistical Computing, Vienna, Austria). The Chi-square test or Fisher’s exact test was applied to analyze the nominal variables, while the T-test was utilized for continuous variables. The decision curve was constructed using “rmda” package and corresponding calibration curves were created using the "rms" package. The variables for the model were selected through stepwise use of Akaike’s information criterion (AIC)^15^ and predictive models used multivariable logistic regression. Receiver operating characteristic curves (ROC) analysis was performed using the “pROC” package. DeLong test was used for statistical comparisons of ROC curves. *P* values less than 0.05 were considered statistically significant.

## Results

### Patient characteristics and laboratory findings

Between January 2019 and December 2021, a total of 3585 patients were hospitalized diagnosed with CAP at our hospital. Excluded from the study were patients with other pathogens infection (n = 2589) and those with other primary diseases in the study (n = 187). A further 629 were excluded because of the absence of complete clinical data or CT examination.

Ultimately, 126 patients from January 2019 to December 2020 were included in the retrospective cohort as a training set, comprising 41 cases of common Mycoplasma pneumoniae pneumonia (CMPP) and 85 cases of RMPP, and 54 patients from January to December 2021 were enrolled in the prospective cohort as a test set, consisting of 17 CMPP and 37 RMPP cases. Consequently,180 MPP patients were enrolled, as illustrated in Supplementary Fig. S1. Differences in clinical and imaging characteristics of patients between the two sets were not statistically significant (see Supplementary Table S1).

The characteristics of the patients in the training and test set on admission are summarized in Table 1. No significant difference was found in gender and age (*P* > 0.05). The clinical symptoms, biochemistry levels, and routine blood markers of RMPP and CMPP within 24 h of admission were compared. Compared with the CMPP group in the training set, differences in the highest temperature within 24 h of admission (*P* < 0.001), HR (*P* = 0.032), AST (*P* = 0.021), ALB (*P* = 0.010), CK (*P* = 0.049), PFD (*P* < 0.001), and PMTD (*P* < 0.001) were statistically significant and the other findings did not differ significantly (*P* > 0.05). In the test set, Hb (*P* = 0.028), LDH (*P* = 0.011), AST (*P* = 0.012), CK (*P* = 0.006) and PFD (*P* = 0.008) were statistically significant between CMPP and RMPP and the other results were not (*P* > 0.05).

**Table 1:** Clinical characteristics of patients in the training and test cohort*.*

Characteristics	Training set			Test set		
	CMPP (n = 41)	RMPP (n = 85)	P value	CMPP (n = 17)	RMPP (n = 37)	P value
Sex (male/female)	21/20	44/41	0.268	12/5	22/15	0.078
Age (years)	4.81 ± 2.79	4.88 ± 2.79	0.889	4.81 ± 2.79	4.64 ± 2.51	0.210
T (°C)	37.32 ± 1.07	38.03 ± 1.28	< 0.001*	37.51 ± 1.30	37.85 ± 1.35	0.388
HR	116.59 ± 17.62	123.74 ± 18.12	0.032*	117.56 ± 14.36	123.32 ± 15.51	0.201
R	25.61 ± 4.94	26.78 ± 3.84	0.079	25.62 ± 3.93	27.03 ± 3.94	0.243
WBC (×10^9^/L)	9.51 ± 4.56	9.71 ± 3.72	0.818	10.25 ± 4.26	9.37 ± 4.66	0.508
NEUT (×10^9^/L)	9.19 ± 4.93	9.18 ± 5.23	0.997	10.12 ± 3.80	9.03 ± 5.01	0.392
Hb (g/L)	128.05 ± 33.63	122.11 ± 9.86	0.257	127.13 ± 7.18	121.32 ± 10.97	0.028*
PLT (×10^9^/L)	277.64 ± 122.79	288.94 ± 129.45	0.625	287.68 ± 119.95	294.73 ± 154.05	0.859
CRP (mg/L)	19.95 ± 18.07	24.94 ± 36.02	0.289	23.00 ± 19.28	24.54 ± 30.54	0.828
LDH (U/L)	354.16 ± 161.08	403.07 ± 167.87	0.108	317.37 ± 100.80	411.19 ± 152.01	0.011*
ESR (mm/h)	31.55 ± 15.24	30.73 ± 18.16	0.786	32.88 ± 12.09	29.54 ± 18.96	0.447
MP-DNA (×10^4^)	539.98 ± 1598.42	1949.96 ± 10,820.71	0.232	249.61 ± 646.86	876.30 ± 21,411.18	0.112
d-dimer (ng/L)	651.84 ± 1532.27	673.84 ± 828.26	0.929	532.31 ± 1098.19	713.05 ± 912.48	0.569
PCT (ng/mL)	0.19 ± 0.37	0.17 ± 0.34	0.786	0.18 ± 0.19	0.13 ± 0.15	0.386
ALT (U/L)	15.66 ± 12.93	22.52 ± 28.57	0.059	15.18 ± 11.32	20.65 ± 17.89	0.188
AST (U/L)	29.43 ± 10.57	35.25 ± 18.01	0.021*	25.94 ± 8.05	34.03 ± 14.37	0.012*
TP (g/L)	68.42 ± 4.64	67.12 ± 5.40	0.154	69.93 ± 4.57	67.78 ± 4.72	0.413
ALB (g/L)	42.73 ± 3.13	40.99 ± 4.37	0.010*	42.02 ± 2.77	41.34 ± 3.21	0.439
CK-MB (U/L)	86.59 ± 71.30	67.59 ± 63.11	0.137	75.75 ± 50.98	65.05 ± 59.70	0.511
CK (U/L)	39.5 ± 47.43	73.71 ± 39.50	0.049*	22.50 ± 8.15	40.08 ± 34.73	0.006*
PFD (days)	5.07 ± 2.71	7.04 ± 4.21	< 0.001*	5.31 ± 2.52	7.92 ± 4.29	0.008*
PCD (days)	9.36 ± 7.08	11.10 ± 6.02	0.326	12.19 ± 9.83	13.49 ± 28.77	0.808
PMTD (days)	2.22 ± 2.33	4.71 ± 3.76	< 0.001*	2.63 ± 3.12	4.05 ± 3.39	0.146

Values are presented as mean ± SD. *T* temperature, *RMPP* refractory Mycoplasma pneumoniae pneumonia, *CMPP* Common Mycoplasma pneumoniae pneumonia, *HR* heart rate,* R* respiratory, *WBC* white blood cell, *NEUT* neutrophil, *Hb* hemoglobin, *PLT* platelets, *CRP* C-reactive protein, *LDH* lactate dehydrogenase, *ESR* erythrocyte sedimentation rate, *PCT* procalcitonin, *ALT* alanine aminotransferase, *AST* aspartate aminotransferase, *TP* Total Protein, *ALB* albumin, *CK-MB* creatine phosphokinase isoenzyme, *CK* creatine kinase, *PFD* preadmission fever duration, *PCD *preadmission cough duration, *PMTD* preadmission macrolides therapy duration.

*P value < 0.05 indicates statistical significance.

### Chest CT quantitative analysis

Quantitative lung features on CT are summarized in Table 2. The RMPP group had a larger volume of pulmonary lesions, and extent of lesions located in the lung of the right upper lobe and right lower lobe compared to the CMPP. The extent of lesions located in the lung of the left lower lobe of the RMPP group was larger than CMPP in the training set (*P* = 0.046), but it was not statistically significant in the test set (*P* > 0.05). The log_2_Φ of the RMPP group was higher than CMPP in two sets (*P* < 0.05).

**Table 2:** Quantitative CT parameters of patients in the training and test set.

Quantitative lung features	Training set			Test set		
	CMPP(n = 41)	RMPP(n = 85)	*P* value	CMPP(n = 17)	RMPP(n = 37)	*P* value
Log_2_Φ	15.63 ± 1.31	16.78 ± 1.08	< 0.001*	15.50 ± 1.26	16.66 ± 1.10	0.002*
Total lung volume (cc)	827.93 ± 368.40	858.92 ± 447.52	0.672	954.93 ± 419.52	799.47 ± 325.31	0.199
Volume of lung infiltration (cc)	71.72 ± 65.43	139.68 ± 104.62	< 0.001*	66.77 ± 74.78	127.96 ± 94.10	0.016*
Percentages of lung infiltration (%)	5.17 ± 5.45	8.98 ± 6.50	< 0.001*	3.65 ± 5.73	8.95 ± 6.46	0.006*
Right upper lobe (%)	37.13 ± 20.78	49.99 ± 21.10	< 0.001*	7.57 ± 13.72	23.71 ± 25.09	0.004*
Middle lobe of right (%)	11.92 ± 24.24	18.87 ± 28.69	0.101	7.79 ± 21.38	18.00 ± 29.22	0.229
Right lower lobe (%)	11.23 ± 18.17	28.30 ± 31.47	0.002*	8.87 ± 17.64	32.69 ± 33.37	0.001*
Left upper lobe (%)	10.51 ± 22.09	19.45 ± 29.49	0.083	6.37 ± 21.71	13.50 ± 23.13	0.269
Left lower lobe (%)	13.44 ± 23.68	22.85 ± 28.39	0.046*	17.46 ± 28.09	17.83 ± 28.49	0.966

*CT* Computed tomography, *CMPP* Common Mycoplasma pneumoniae pneumonia, *RMPP* refractory Mycoplasma pneumoniae pneumonia.

*P value < 0.05 indicates statistical significance.

### Logistic regression and nomogram

Overfitting poses a notable challenge in modeling, resulting from the inclusion of an excessive amount of model parameters. To address this issue, a backward stepwise procedure was utilized, employing the AIC to select variables for the clinical models, penalizing those models with an abundance of parameters. The most influential variables were identified through a stepwise approach, systematically excluding variables until the optimal model fit was achieved. PFD, PMTD, temperature, and AST were included as predictors in the clinical model, Log_2_Φ was included in the imaging model and all these five predictors were included in the clinical-imaging model (Table 3).

**Table 3:** Multiple models with a combination of clinical and imaging parameters by stepwise logistic regression analysis predicting the RMPP.

	Variable	β	OR	95% CI for OR
Clinical model	T	0.547	1.728	1.203, 2.483
	AST	0.026	1.026	0.987, 1.066
	PFD	0.149	1.161	0.997, 1.351
	PMTD	0.342	1.408	1.179, 1.680
Imaging model	Log_2_ Φ	0.853	2.347	1.642, 3.532
Clinical-imaging model	Log_2_ Φ	1.421	4.141	2.251, 7.621
	T	0.578	1.782	1.164, 2.728
	AST	0.017	1.018	0.972, 1.067
	PFD	0.085	1.089	0.916, 1.294
	PMTD	0.571	1.768	1.385, 2.258

*PFD* preadmission fever duration, *PMTD* preadmission macrolides therapy duration, *OR* odds ratio.

Clinical model including variables selected with stepwise method using the AIC.

Imaging model including Log_2_ Φ.

Clinical-imaging model including Clinical model variables and Log_2_ Φ.

Variables were presented in the model by using the following formula:

*Clinical model:*

Probability of RMPP × 100 = − 2272 + 54.7 T + 2.6AST + 14.9PFD + 34.2PMTD.

*Imaging model:*

Probability of RMPP × 100 = − 1315 + 85.3Log_2_Φ.

*Clinical-imaging model:*

Probability of RMPP × 100 = − 4709 + 142.1Log_2_Φ + 57.8 T + 1.7AST + 8.5PFD + 57.1PMTD.

**Figure 2:**
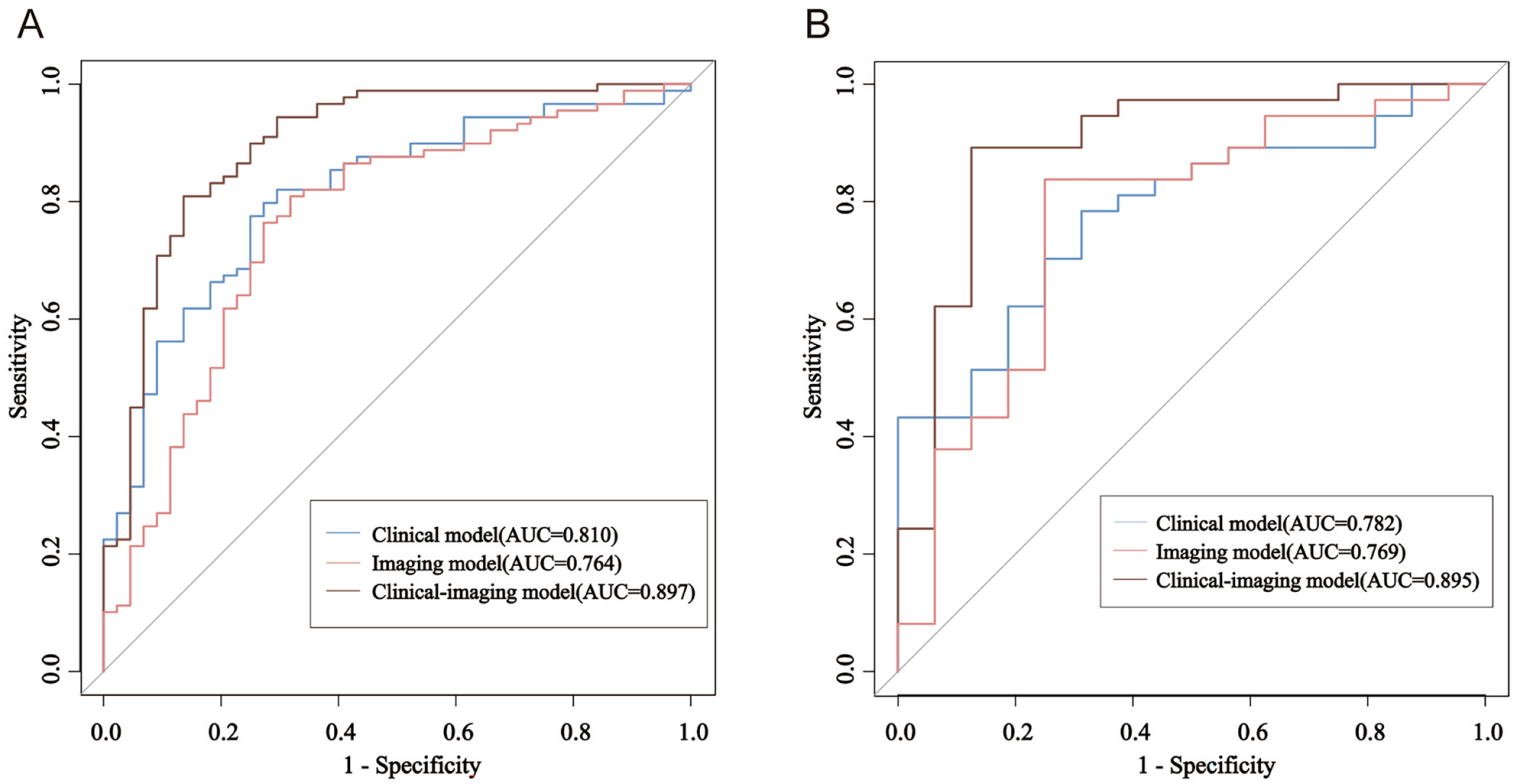
Receiver operating characteristic (ROC) and areas under the ROC curve (AUC). (**A**) ROC analysis showed an AUC of 0.897 for the clinical-imaging prediction model, an AUC of 0.810 for the clinical prediction model, and an AUC of 0.764 for the imaging prediction model in the training set, suggesting that the combined predictor has a greater diagnostic value than clinical data or imaging data alone. (**B**) ROC curves of the clinical prediction model, the imaging prediction model, and the clinical-imaging prediction model in the test set showed the AUC were 0.895, 0.769 and 0.782 respectively.

The ROC of the training set was plotted in Fig. 2A with AUC values of 0.810, 0.764, and 0.897, and the test set was plotted in Fig. 2B with AUC values of 0.782, 0.769, and 0.895 respectively. The AUC value of the Clinical-imaging model (0.897, 95% CI 0.835, 0.957) was significantly higher than the Clinical model (0.810, 95% CI 0.734, 0.887, P = 0.012) and the imaging model (0.764, 95% CI 0.675, 0.854, P < 0.000). Independent verification conducted in the validation cohort demonstrated enhanced performance with clinical-imaging markers yielding a statistically significant AUC value of 0.895 (95% CI 0.789, 1.000). In the training set, the Clinical-imaging model exhibited a positive predictive value of 85.29% and a negative predictive value of 84.85%, while in the test set, these values were 78.57% and 87.18%, respectively.

**Figure 3:**
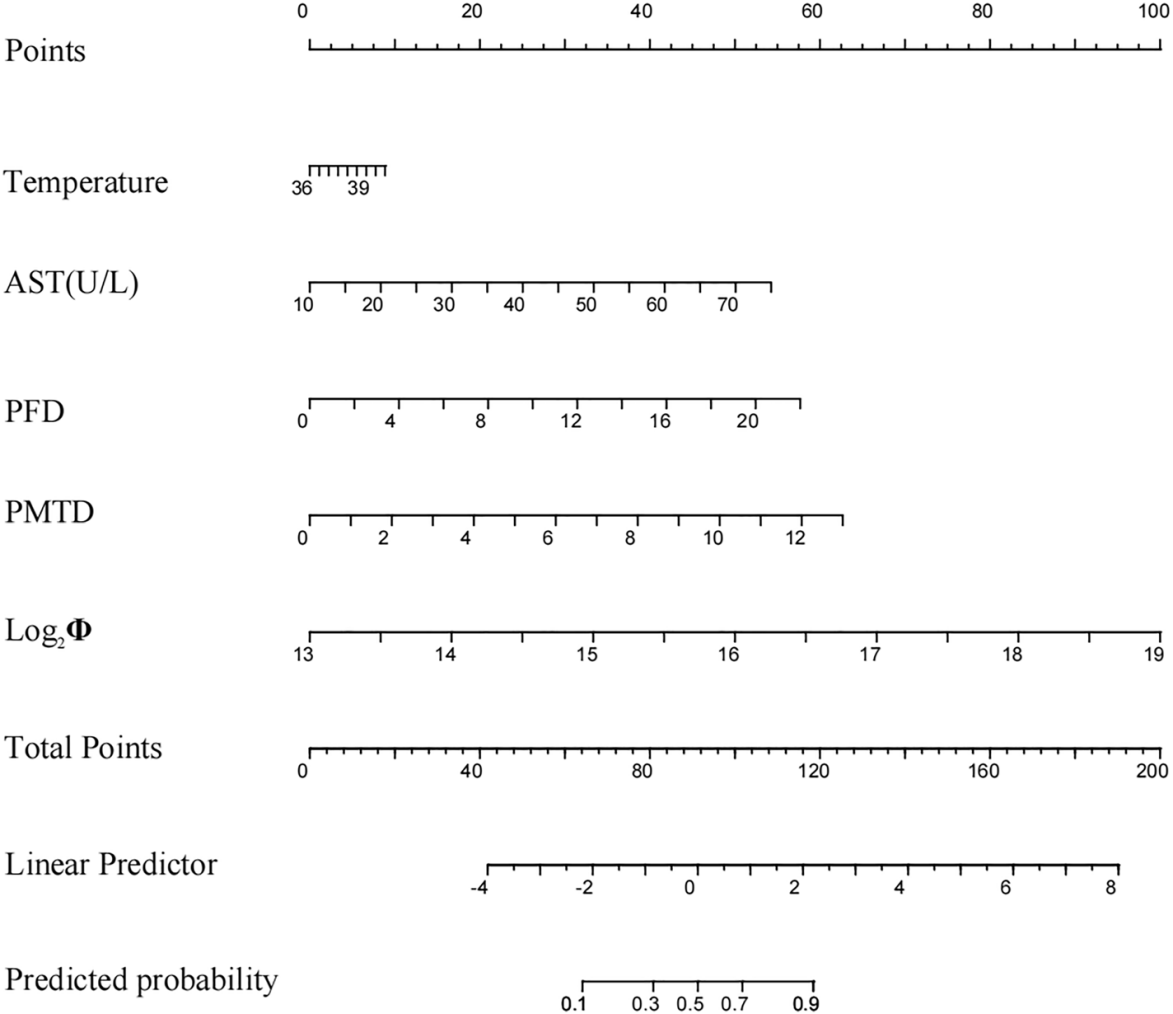
Nomogram. The nomogram was built in the training set with the temperature, AST, PFD, PMTD and Log_2_Φ. *AST* aspartate aminotransferase, *PFD* preadmission fever duration, *PMTD* preadmission macrolides therapy duration.

A nomogram was constructed by incorporating the predictors in the clinical-imaging model (Fig. 3). The nomogram was created by giving each independent influencing element a weighted score. The highest score is 120 points, and the range of RMPP incidence is 0.1–0.9. A higher chance of occurrence is indicated by a larger score obtained by adding the distribution points of each high-risk factor in the nomogram.

### Model evaluation

**Figure 4:**
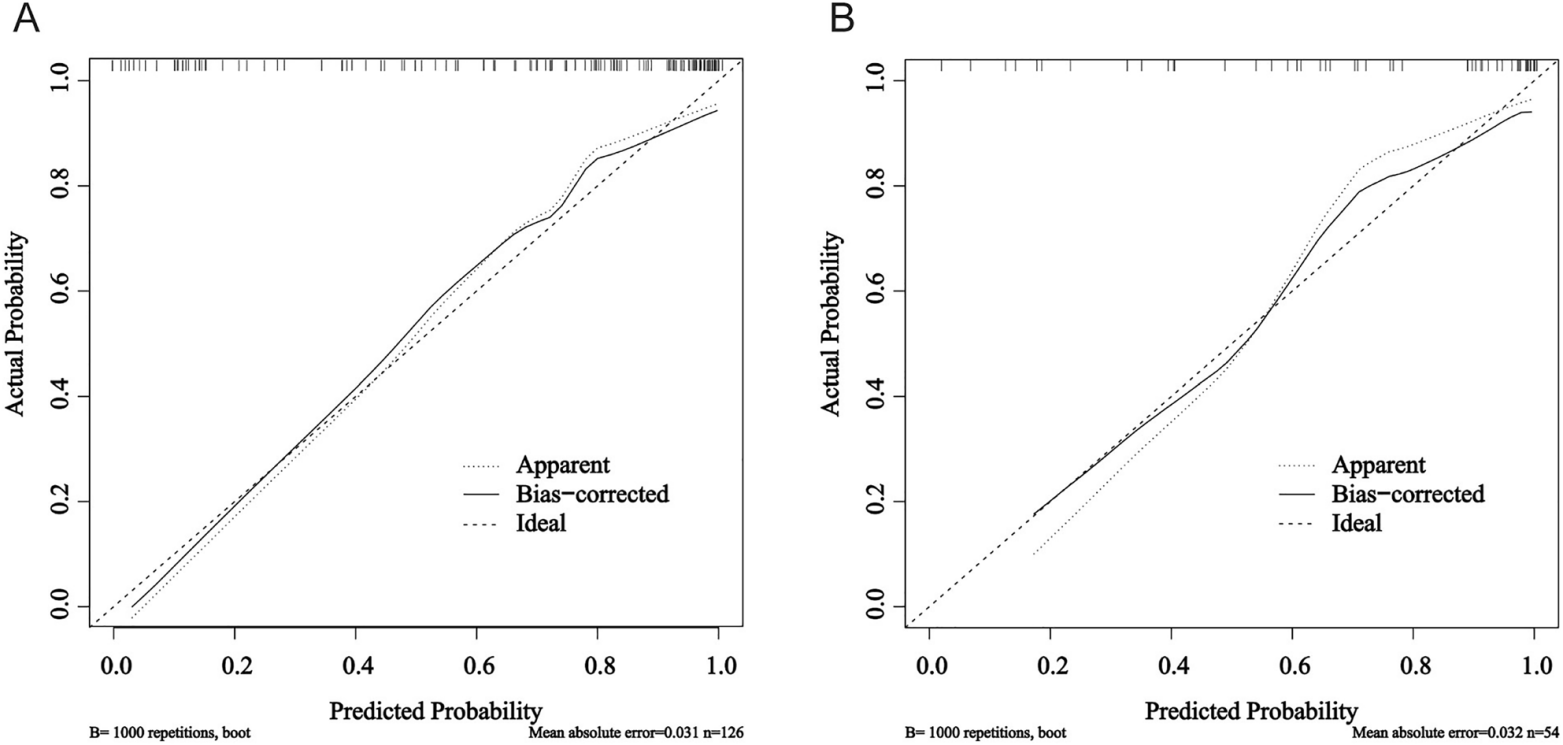
Calibration curve of the clinical-imaging prediction model. The calibration curve was determined with bootstrap analysis to get bias-corrected estimation. The y-axis shows the actual result. The x-axis represents the predicted probability of refractory mycoplasma pneumoniae pneumonia. Ideal represents the ideal curve, apparent represents the uncalibrated prediction curve and Bias-corrected represents the calibration prediction curve. (**A**) The calibration curve of the training set. (**B**) The calibration curve of the test set.

Figure 4A,B present graphical representations of calibration of the training set and the test set individually, the clinical-imaging prediction model (both apparent and bias-corrected) was close to ideal calibration.

**Figure 5:**
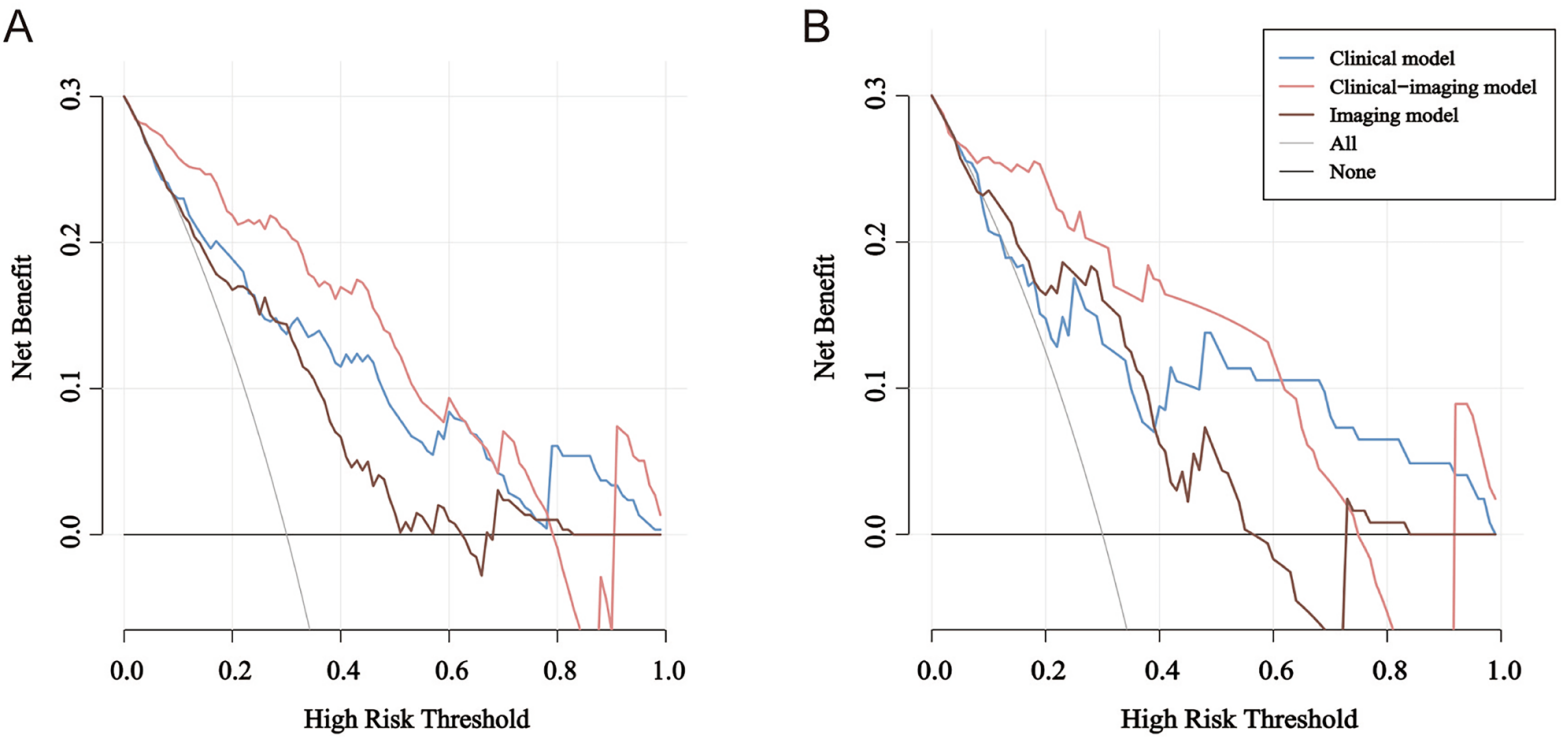
The decision curves. The y-axis measures the net benefit. The blue solid line, grey solid line, and red solid line represent the net benefit of the clinical model, the imaging model, and the clinical-imaging model at different threshold levels, respectively. (**A**) DCA for three models in the training set. (**B**) DCA for three models in the test set.

We subsequently compared the clinical performance of the integrated model to the clinical model and imaging model separately using decision curve analysis, which is based on the idea of net benefit and considers the impact of potential benefit and potential harm from the use of predictive models. Figure 5A,B illustrate the decision curves for the three models to predict the correct diagnosis of RMPP in the training set and test set. The decision curve analysis graphically shows the clinical usefulness of each model based on a continuum of potential thresholds for diagnosis of RMPP risk (x-axis) and the net benefit of using the model to risk stratify patients (y-axis) relative to assuming CMPP. In this analysis, the clinical-imaging model provided a larger net benefit across the range of diagnosis of RMPP compared with the other two models.

## Discussion

MP is a significant pathogen responsible for a notable percentage of CAP cases in children under the age of 18^21,22^. Although macrolide antibiotics are useful in treating MP infection, RMPP is on the rise due to increased macrolide use and the emergence of antibiotic-resistant bacteria^10,23^. RMPP typically induces a prolonged fever that may advance to severe, potentially fatal pneumonia^9^ and compared to CMPP, patients with RMPP usually require a longer course of antibiotics and higher doses of glucocorticoids^24,25^. In recent years, tetracyclines seem to be uplifting and promising in the treatment of RMPP^26^. A prediction model can help identify RMPP at an early stage and consider a change in antibiotic prescription.

Previous studies on predicting RMPP have predominantly concentrated on serological markers. Jun Wen et al. identified serum ferritin, d-dimer, and CRP as important predictors of RMPP^7^. Yaoyao et al. highlighted neutrophil/lymphocyte ratio, mean platelet volume/lymphocyte ratio as key predictors^27^. Currently, the visualized characters from CT scans have hardly been studied as potential markers for RMPP. The pathogenesis of RMPP is considered related to MP invasion and immune dysfunction^28^. MP infects the body and causes the expression of respiratory epithelial adhesion protein^29^. Simultaneously, excessive inflammation in the lungs and the whole body occurred following MP infection^30^. Compared with children with CMPP, RMPP is easier to complicate with pleural effusion and atelectasis Chest radiographs and CT scans can be particularly helpful in determining regional density distribution as well as the extent and form of lung injury. Lung lesion severity can assist doctors in differentiating RMPP. In contrast to the reporting with the visual quantitative estimation of lesions provided by a radiologist, AI analysis of radiologic images possesses good reproducibility and reduces the radiologist’s reading time. To the best of our knowledge, Weihong Lu et al. apply quantitative CT scoring to the chest imaging assessment of MPP, which was the latest study of radiological scoring tools for predicting RMPP^14^. However, they require radiologists or clinicians to determine CT scores on the image, which is time consuming. The quantitative assessment of CT features of MPP by AI software has not been reported extensively. In this study, we analyzed the CT scans of MPP patients using the AI Intelligent Assistant Analysis System. This system can precisely segment different anatomical features of the lungs to discover infection regions and calculate the proportion of infection in both lungs, as well as their lobes and segments. Studies have shown that the use of autonomous 3D AI software for quantitative assessment of radiological findings can predict clinical deterioration or mortality in patients with COVID-19 pneumonia^31^. Additionally, this AI-based software has been found to surpass the diagnostic accuracy of experienced human specialists in detecting pleural effusion and interstitial lung disease^32^.

The degree of the pulmonary lesion was a reliable feature for assessing RMPP^33^, Lobar pneumonia and large pleural effusion in MPP, suggest a more severe disease state possibly attributed to macrolide resistance, increased MP burden, intense host inflammatory responses, or other contributing factors. However, we have no specific indicator to quantify this correlation. In this study, we defined a constant Φ that was the summation of the CT value per unit volume of lesion quantified by AI automatically. Then, lesion volume and Log_2_Φ were compared between RMPP and CMPP in the training set. The analysis revealed that RMPP patients exhibited a higher lesion volume and Log_2_Φ value. We additionally confirmed this phenomenon in another test set. Log_2_Φ, a constant derived from the combination of lesion volume and CT value in each patient, could be identified as a prognostic indicator. In addition, four clinical prognostic factors were identified, including the highest temperature on the first day of admission, AST, PMTD, and PFD, to predict the occurrence of RMPP and reduce the incidence of complications and long-term pulmonary damage. Although the etiology of RMPP is unknown, it is largely assumed that inflammatory and immunological responses have a role in the progression of the disease. Some biomarkers linked to inflammatory reactions had been used to predict RMPP by previous studies^33,34^. The peak temperature in the RMPP group was significantly higher than that in the CMPP group on the first day of admission^35^. These markers were higher in the RMPP group than in the CMPP group, implying that children with refractory M. pneumoniae pneumonia have more pronounced inflammatory responses. After an MP infection, hepatic impairment is common extrapulmonary damage^34^. The study showed that the AST in the RMPP group was higher^35^. Macrolides have long been thought to be the best treatment for M. pneumoniae pneumonia (MP) in children^36^. Based on physicians ' clinical experience, most patients had already received macrolides therapy before admission, but they are still hospitalized because of poor response to macrolides. In this study, we found that the longer the macrolides therapy duration before admission they took, the greater the risk of RMPP.

Finally, we integrated the Log_2_Φ and clinical predictive factors to obtain a better comprehensive imaging-clinical prediction model. The sensitivity and specificity of the training set were 0.809 and 0.864, the AUC was 0.897 and the sensitivity and specificity of the test set were 0.892 and 0.875, the AUC was 0.895. This research is pioneering in its combination of clinical biomarkers and automated AI-driven CT quantification to predict RMPP. Our study demonstrated that the integration of Log_2_Φ with clinical biomarkers including temperature, AST, PMTD, and PFD could effectively predict the likelihood of MPP patients progressing to RMPP.

The novelty of our proposed method lies in the following aspects. First, quantitative CT analysis using automated quantification takes only a few seconds to achieve complete segmentation of lung lesions with nearly flawless reproducibility, whereas manual scoring is a more time-intensive process with only moderate reproducibility. This preliminary study confirms the suitability of available AI-based segmentation tools for analyzing MPP data, to support the diagnosis and quantification of MPP lung lesions. Second, our proposed constant, Φ, based AI-aided diagnosis system is a novel approach to quantitatively evaluate pulmonary lesions of MPP patients' CT scans. This is not seen in the literature up to this point. Third, to our knowledge, it is the first study to apply automated AI-driven CT quantification combined with clinical parameters that can diagnose RMPP earlier.

Inevitably there are assumptions and limitations in the models. First, initially, we analyzed CT scans obtained within 48 h of diagnosis. Due to the variability in radiological findings throughout the disease progression, our models were not trained on data representative of the intermediate and late stages of the disease. Second, because the data for the study was only taken from the baseline CT and one single-center CT, multi-center validation with a bigger sample size is needed to assess the possible benefit of AI software in MPP patient care.

In conclusion, this study presented an imaging-clinical prediction model that incorporates imaging and clinical risk indicators and may be utilized to quickly identify RMPP and CMPP, laying the groundwork for early clinical and accurate therapy for RMPP.

### Supplementary Information

Supplementary Information.

## Data Availability

The datasets used and/or analyzed during the current study can be obtained from the corresponding author on reasonable request.
