# Supplementary material for: Model based on the automated AI-driven CT quantification is effective for the diagnosis of refractory Mycoplasma pneumoniae pneumonia
Source: Sci Rep. 2024 Jul 13;14:16172. doi: 10.1038/s41598-024-67255-8 (PMC11246496; doi:10.1038/s41598-024-67255-8)
Supplement: Supplementary file 1 — Supplementary Information. [file 41598_2024_67255_MOESM1_ESM.pdf]

Supplementary Information

Model based on the automated AI-Driven CT quantification is effective for the diagnosis of refractory Mycoplasma pneumoniae pneumonia

Yali Qian[1, +], Yunxi Tao[2, +], Lihui Wu [1], Changsheng Zhou[3], Feng Liu[4], Shenglong Xu[2], Hongjun Miao[1], Xiucheng Gao[5, *], and Xuhua Ge[1, *]

[1] Department of Emergency / Critical Medicine, Children's Hospital of Nanjing Medical University, Nanjing, Jiangsu, China

[2] School of Pediatrics, Nanjing Medical University, Nanjing, Jiangsu, China

[3] Department of Diagnostic Radiology, Jinling Hospital, Medical School of Nanjing University, Nanjing, Jiangsu, China

[4] Department of Respiratory Medicine, Children's Hospital of Nanjing Medical University, Nanjing, Jiangsu, China

[5] Department of Radiology, Children's Hospital of Nanjing Medical University, Nanjing, Jiangsu, China

[*]Corresponding.gexuhua@njmu.edu.cn; gausli@163.com

[+] These authors contributed equally: Yali Qian and Yunxi Tao

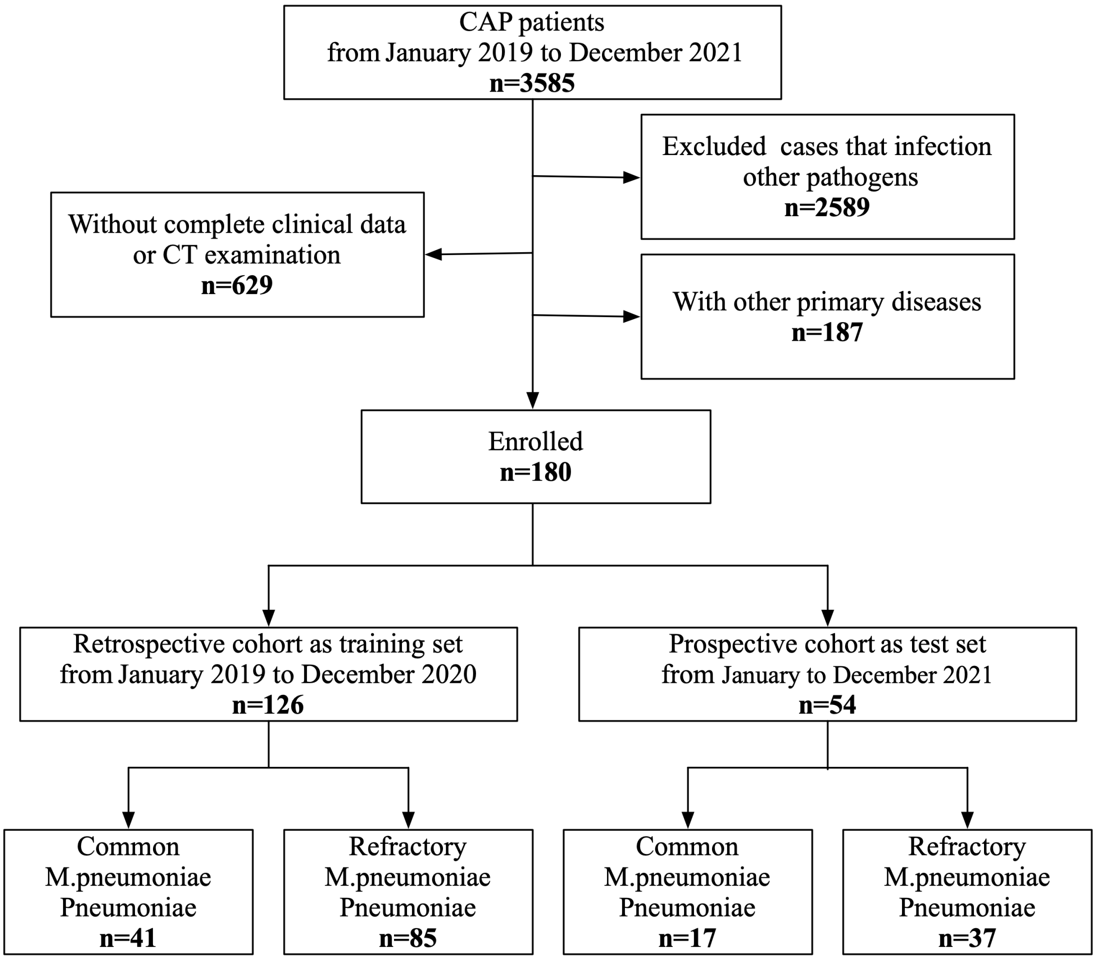

Supplementary Figure S1. Study flow. *CAP* community-acquired pneumonia, *CT* computed tomography.

	**Training set**	**Test set (n=54)**	***P* value**
Sex(male/female)	65/61	34/20	0.157
Age(years)	4.86±2.78	5.00±2.77	0.742
T (°C)	37.80±1.29	37.74±1.33	0.811
HR	121.37±18.20	121.58±15.27	0.937
R	26.41±3.75	26.60±3.95	0.755
WBC(*10^9/L)	9.65±4.80	9.64±4.52	0.987
NEUT(*10^9/L)	9.18±5.12	9.35±4.67	0.822
Hb (g/L)	124.07±21.00	123.08±10.26	0.665
PLT(*10^9/L)	285.20±126.92	292.20±143.49	0.744
CRP (mg/L)	23.29±31.25	24.07±27.45	0.866
LDH (U/L)	386.89±166.65	382.87±144.29	0.869
ESR (mm/h)	31.00±17.19	30.54±17.12	0.871
MP-DNA (*10^4)	1773.38±9373.91	687.12±1838.22	0.204
D-dimer (ng/L)	666.56±1105.57	658.49±965.06	0.961
PCT (ng/mL)	0.18±0.35	0.15±0.16	0.416
ALT (U/L)	20.25±24.67	19.00±16.28	0.687
AST (U/L)	33.33±16.12	31.58±13.25	0.449
TP (g/L)	67.55±5.17	68.12±4.66	0.467
ALB (g/L)	41.57±4.07	41.54±3.07	0.960
CK-MB (U/L)	73.87±66.26	68.28±56.93	0.565
CK(U/L)	59.77±40.99	35.39±30.34	0.051
PFD (days)	6.39±3.88	7.13±4.01	0.253
PCD (days)	10.88±6.42	13.09±24.52	0.547
PMTD (days)	3.88±3.54	3.62±3.35	0.634
**Quantitative lung features**			
$Log_2Φ$	16.40±1.28	16.29±1.27	0.595
Total lung volume(cc)	848.66±421.83	546.40±359.47	0.971
Volume of lung	117.19±98.59	109.48±92.45	0.615
Percentages of lung	7.72±6.41	7.35±6.67	0.735
right upper lobe (%)	23.79±2.85	18.84±2.33	0.225
middle lobe of right	17.08±2.74	15.82±2.73	0.785
right lower lobe (%)	22.65±2.88	25.50±3.13	0.569
left upper lobe (%)	16.61±2.75	11.56±2.27	0.207
left lower lobe (%)	19.88±2.72	17.72±2.81	0.634

Supplementary Table S1. Clinical and imaging characteristics of patients in the training and test set. Values are presented as mean ± SD. *T* temperature, *RMPP* refractory Mycoplasma pneumoniae pneumonia, *CMPP* Common Mycoplasma pneumoniae pneumonia, *HR* heart rate, *R* respiratory, *WBC* white blood cell, *NEUT* neutrophil, *Hb* hemoglobin, *PLT* platelets, *CRP* C-reactive protein, *LDH* lactate dehydrogenase, *ESR* erythrocyte sedimentation rate, *PCT* procalcitonin, *ALT* alanine aminotransferase, *AST* aspartate aminotransferase, *TP* Total Protein, *ALB* albumin, *CK-MB* creatine phosphokinase isoenzyme, *CK* creatine kinase, *PFD* preadmission fever duration, *PCD* preadmission cough duration, *PMTD* preadmission macrolides therapy duration.
